# Royal Medical and Chirurgical Society

**Published:** 1899-01-14

**Authors:** 


### ROYAL MEDICAL AND CHIRURGICAL SOCIETY.

At the meeting last Tuesday another old belief was very effectually exploded, namely, the idea that aneurism of the aorta causes hypertrophy of the left ventricle of the heart. A paper was read by Dr. James Calvert in which the whole subject was considered anew in the light of a careful examination which he had made of the post-mortem records of St. Bartholomew's Hospital—a veritable mine of pathological lore—and the result was to show that, although it was true that in about half of the cases of aneurism some thickening of the ventricle was found, there was nearly always present some one or more of the recognised causes of this condition.

Dr. Calvert said that some authorities stated that aortic aneurism was certainly a cause of hypertrophy of the left ventricle, while others were equally positive that it was not, and he pointed out how embarrassing to the teacher and how confusing to the student this conflict of opinion was. He had, therefore, thought it worth while to see what light the post-mortem records of the hospital threw upon the subject. In doing this, his work had been greatly lightened by reference to Dr. Oswald Browne's recent work on "Aneurysms of the Aorta," which was based upon an analysis of all cases of aneurism of the aorta dying in St. Bartholomew's Hospital during the last thirty years upon whom an examination had been made after death and its results carefully recorded. Stated briefly, Dr. Calvert gave the results of his research as follows: The cases dealt with numbered 124, all of which were cases of aneurism of the arch of the aorta. In 68 of them there was no hypertrophy of the left venticle; in 47 of them the hypertrophy could be perfectly explained by other causes present; in nine of them the hypertrophy could be very probably explained by other causes present. Therefore, so far as the records of St. Bartholomew's Hospital were concerned, there was practically no evidence in favour of aortic aneurism being a cause of hypertrophy of the left ventricle. The origin of the hypertrophy in those cases in which it existed was in a large number of cases traced by the author of the paper to the presence of one or other of its recognised causes, especially to the arteriocapillary fibrosis which occurs with kidney disease, and to some greater or less degree of atheroma.

Some little discussion took place in regard to the question whether atheroma could be properly looked upon as a cause of hypertrophy of the ventricle, and it was even maintained by one speaker that rigidity of the aorta did not increase the work of the heart. At this time of day it seems hardly worth while to discuss such a suggestion, but as was pointed out, if we remember rightly, by Dr. Rolleston, it did not much matter, so far as the question under discussion was concerned, for whether atheroma arose in consequence of increased peripheral resistance, or from over-violent action of the heart, as in labourers and athletes, in either case it indicated pre-existing strain of the aorta, and strain on the aorta meant work in the heart, and work in the heart lead to hypertrophy, so that without in any way maintaining that atheroma caused hypertrophy, the existence of atheroma was quite sufficient to show that the conditions provocative of hypertrophy had existed. It is curious, however, to note how slight is the hypertrophy which sometimes co-exists with considerable atheroma, and we think it might have been well to have entered more fully upon the question how far hypertrophy may vanish and end even in atrophy of the heart, a condition which Mr. Stephen Paget mentioned as sometimes being met with in aneurism. Perhaps if cases of aneurism died early, before long invalidism had led to shrinking of the hypertrophy, this latter condition would be found still more often then than is the case. But even so the aneurism would not be its cause. A very practical bearing was given to the whole discussion by the remarks of Dr. Norman Moore, who pointed out that as a corollary of Dr. Calvert's views—views which he had long taught—we must recognise that the presence of hypertrophy of the heart in any case of aneurism must be taken to indicate the presence of some complication. If we find hypertrophy of the left ventricle we have to do with something else besides aneurism, possibly some latent kidney trouble, and one need hardly say that this may have an important bearing upon both the prognosis and the treatment of the case.

